# BileActome reveals community-assembled bile acid metabolism in the rumen microbiome

**DOI:** 10.1093/ismeco/ycag205

**Published:** 2026-07-14

**Authors:** Boyan Zhang, Xianzhe Jiang, Hailong Zhao, Bing Wang

**Affiliations:** State Key Laboratory of Animal Nutrition and Feeding, College of Animal Science and Technology, China Agricultural University, Beijing 100193, China; State Key Laboratory of Animal Nutrition and Feeding, College of Animal Science and Technology, China Agricultural University, Beijing 100193, China; State Key Laboratory of Animal Nutrition and Feeding, College of Animal Science and Technology, China Agricultural University, Beijing 100193, China; State Key Laboratory of Animal Nutrition and Feeding, College of Animal Science and Technology, China Agricultural University, Beijing 100193, China; Sanya Institute of China Agricultural University, Sanya 572000, China

**Keywords:** bile acids, metagenome-assembled genomes, bile salt hydrolase, *Bai* operon, hydroxysteroid dehydrogenases

## Abstract

Microbial bile acid metabolism is an important link between microbiomes and host physiology, but its genetic basis remains difficult to resolve from genome and metagenome data. This is largely because existing annotation resources are not designed for the high sequence diversity and functional complexity of microbial bile acid genes. Here we present BileActome, a reusable annotation resource developed specifically for microbial bile acid metabolism. BileActome defines 27 experimentally supported gene families, including bile salt hydrolases, bile acid–inducible operon genes, and microbial hydroxysteroid dehydrogenases. Its design prioritizes experimentally supported functional sites when available and conserved domain features otherwise, while also distinguishing key functional subtypes. We applied BileActome to 1693 high-quality rumen metagenome-assembled and isolate genomes and validated its performance using controlled *in vitro* rumen fermentations under three bile acid interventions. In metagenomic gene-catalog analyses, BileActome enabled pathway-level interpretation of microbial responses to bile acid exposure, with the most reproducible responses centered on Bai-associated gene families. At genome scale, it generated a phylogeny-informed map of bile acid metabolic potential that was broader and more informative than Kyoto Encyclopedia of Genes and Genomes (KEGG)-based annotation. Further analyses of genomes, local gene organization, and genome-level guilds showed that bile acid metabolism in the rumen is modular, phylogenetically structured, and distributed across different microbial members. Deconjugation and oxidation/epimerization-related functions were widespread, whereas complete bile acid–inducible systems were less common. Together, these findings support a community-assembled model of bile acid metabolism and establish BileActome as an open and reproducible framework for studying specialized microbial functions in complex ecosystems.

## Graphical Abstract

**Figure:**
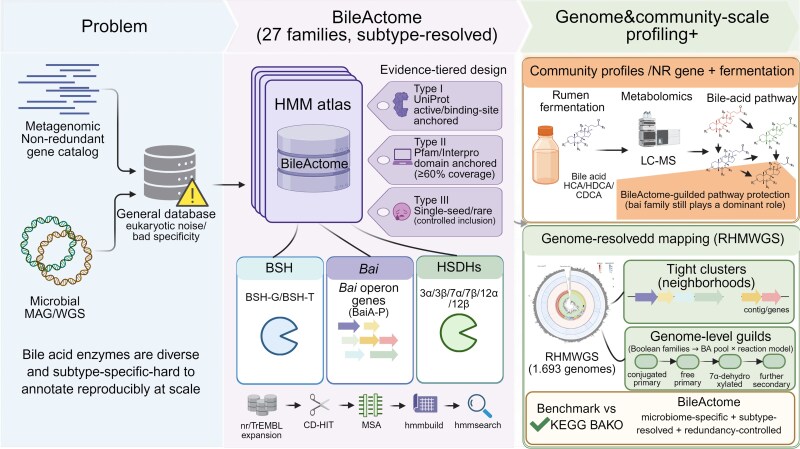

Created in BioRender. Zhang, B. (2026) https://BioRender.com/ydlwbf4

## Introduction

Microbial bile acid metabolism is an important link between microbial communities and host physiology. It is mediated mainly by bile acid–inducible (*Bai*) operon genes, bile salt hydrolase (*BSH*), and hydroxysteroid dehydrogenase (*HSDH*), and influences nutrient absorption, immune regulation, and community structure [1, 2]. Defining how this metabolic capacity is distributed across microbial communities is therefore essential for understanding how microbiome composition relates to biological function. In our recent study, we showed for the first time that the rumen microbiome can convert primary bile acids into secondary bile acids and related derivatives [3]. However, Kyoto Encyclopedia of Genes and Genomes (KEGG)-based annotation did not detect clear changes in the corresponding microbial bile acid metabolic genes, highlighting a gap between metabolite-level observations and gene-level interpretation.

Several existing resources can support the study of bile acid metabolism, but each has important limitations. MetaCyc provides well-curated biochemical reactions, yet its coverage of microbial bile acid metabolism is uneven, which limits its use as a complete reference [4]. MiMeDB 2.0 is useful for metabolite-centered interpretation, but it is better suited to downstream contextual analysis than to upstream gene discovery or standardized gene quantification [5]. KEGG remains the most widely used reference for functional annotation, but as a broad general-purpose resource, it does not resolve microbial bile acid metabolism in sufficient detail [6, 7]. More recently, hidden Markov model (HMM)–based approaches have shown promise for identifying bile acid–related genes. However, these efforts have covered only part of the known gene space and have generally not been released as stable, reusable annotation resources with standardized workflows [8].

To address these limitations, we developed BileActome, a reusable functional annotation database designed specifically for microbial bile acid metabolism. BileActome uses an evidence-tiered construction strategy that prioritizes experimentally supported active or binding sites when available [9–11]. It also captures biologically meaningful subtype structure, including substrate-specific *BSH* subtypes and position- or cofactor-specific *HSDH* families [12–16]. In addition, BileActome is provided with open and standardized workflows that support two common microbiome applications: genome-resolved annotation of metagenome-assembled genomes (MAGs) and isolate whole-genome sequences (WGSs), and community-level profiling based on nonredundant gene catalogs, which intends to provide a general annotation framework for microbial bile acid metabolism and may be applicable to all host-associated microbiome systems with bile acid metabolic capacity or potential.

## Materials and methods

### Construction of BileActome: a curated functional annotation database of bile acid metabolism

To identify and annotate bile acid–metabolizing genes from microbiomes, a combination of curated database mining, sequence homology search, protein domain annotation, and HMM construction [17] was applied targeting three major gene families: *BSH* [15], *Bai* [18], and *HSDH* [12]. Based on available annotation evidence, the bile acid–metabolizing genes were categorized into three types: Type I: Genes with experimentally validated active sites and/or binding sites annotated in UniProt. Type II: Genes without verified active and/or binding sites but with reliable predicted functional domains identified via InterProScan. Type III: Genes lacking both functional site annotations and sufficient homologs for prediction, typically only having a single reference sequence.

For Type I genes, amino acid sequences were collected from the UniProtKB/Swiss-Prot database using gene-specific queries, ensuring that only reviewed entries were included [19]; we call these reviewed entries the core sequence (Supplementary Table S1). Among them, *BSH* is divided into two types: taurine-based (*BSH-T*) and glycine-based (*BSH-G*). *3α-HSDH* is divided into three types: NAD^+^-dependent (*3α-HSDH-NADH*), NADP^+^-dependent (*3α-HSDH-NADPH*), and steroidal substrate-specific(*3α-HSDH-steroid*). *3β-HSDH* is divided into two types: NAD^+^-dependent (*3β-HSDH-NADH*), NADP^+^-dependent (*3β-HSDH-NADPH*). *7α-HSDH* is divided into three types: NAD^+^-dependent (*7α-HSDH-NADH*), NAD^+^ with nonsteroidal substrate (*7α-HSDH-NADH-NS*), and NADP^+^-dependent (*7α-HSDH-NADPH*). The remaining Type I families, including *7β-HSDH*, *BaiA1/A2*, *BaiCD*, *BaiF*, *BaiG*, *BaiH*, *BaiI*, and *BaiK*, were not further subdivided by substrate or cofactor dependence. These core sequences were used as anchors to collect homologous sequences and ensure inclusion of validated gene representatives. Using each core sequence as query, unverified homologous sequences that were collected from NCBI nr and UniProt TrEMBL databases were retrieved through BLASTp [20]. Redundant sequences were removed using CD-HIT [21, 22] and were propagated to homologous sequences using MUSCLE v5 [23] multiple sequence alignment. Manually annotated active and/or binding sites is strictly preserved (100%) when BLASTp, CD-HIT, and MUSCLE runs.

For Type II genes lacking validated sites but containing predictable domains including *BaiB, BaiE*, and *BaiN*, the following steps were applied:

1. Using each core sequence as query (Supplementary Table S1), homologous sequences were retrieved through BLASTp [20] against the NCBI nr and UniProt TrEMBL databases. Redundant sequences were removed using CD-HIT (threshold: 95% identity) [21, 22], which would generate *_cd_hit.fasta
2. InterProScan annotation: Uploading the *_cd_hit.fasta to InterProScan (v5) [24], InterProScan was used to annotate Pfam domains [25, 26]. For domain-based sequence integration, only those domain segments with ≥60% alignment coverage relative to the Pfam model were retained for further analysis, ensuring structural and functional 90% relevance of conserved domains [27–29].
3. Domain alignment: The corresponding Pfam *.seed files were converted to FASTA format, and domain-containing sequences were aligned to seed alignments using MUSCLE [23].
4. Extraction of domain sites: Domain-aligned sequences were analyzed to identify conserved residues, and position-specific annotations were stored.
5. Final domain alignment: All domain-aligned sequences and core sequences were merged into a unified multiple sequence alignment (MSA) file with preserved domain positions.

For Type III genes with no annotation and only few core sequences available (*BaiJ, BaiO, BaiP*, and *12α-/12β-HSDH*), the core sequence was directly used for model building without additional alignment or annotation.

All aligned sequence files (from Type I/II/III) were converted to Stockholm format, which also embeds site annotations (if available). The final HMM profiles were constructed using hmmbuild function of HMMER [17]. At this point, the BileActome library has been constructed, and the 27 genes included in BileActome can be searched and compared directly in the whole microbial genome and the metagenome assembled genome through the hmmsearch function of HMMER. By customizing the script, we can directly extract the sequence corresponding to the search result. According to the recommendations of the HMMER official manual (E-value ≤1 × 10^−3^) [17].

### Construction of a rumen high-quality metagenome-assembled genomes and whole-genome sequences

We collected all currently published rumen MAG up to February 2025 [3, 30–42], together with isolate WGS from the Integrated Microbial Genomes (IMG) database and newly generated MAG in this study, resulting in a total of 36 041 raw MAG/WGS genomes. To annotate bile acid metabolism genes in isolated and cultured rumen microorganisms, we used the “Genomes by Ecosystem” module search under the “Genome Browser” function of the IMG system to search for keywords in the “Specific Ecosystem” category microorganisms belong to “rumen” and “rumen fluid” [43]. The quality of the raw MAG/WGS was assessed using CheckM (v.1.0.7) [44], MAGs/WGSs with over 95% completeness and <5% contamination were selected for further analysis. High-quality MAGs/WGSs underwent dereplication using dRep software [45]. Ultimately, 1693 RHMWGSs were obtained. After filtering, RHMWGSs underwent annotation using GTDB-Tk (v2.6.1) [46] based on the Genome Taxonomy Database. For the phylogenetic analysis, we used PhyloPhlAn (v.3.1.68) to build a maximum-likelihood phylogenomic tree and tvBOT [47] for visualization.

### *In vitro* rumen fermentation

Refer to the previous research [3], rumen fluid was obtained from six ~6-month-old Hu sheep fitted with fistulas. Before their morning feeding, mixed rumen contents were collected and passed through four layers of cheesecloth to filter the rumen fluid into a graduated cylinder. Throughout the collection process, CO₂ was continuously bubbled to maintain anaerobic conditions. A buffer solution with a pH of 6.87 was prepared as per the previous methodology, with CO₂ also continuously infused into the buffer for ~30 min prior to inoculation [48]. Cysteine hydrochloride was added to the buffer to serve as a chemical-reducing agent. For each incubation glass bottle (with a capacity of 120 ml), 0.5 g of substrates (Supplementary Table S2), 15 ml of filtered rumen fluid, and 30 ml of pre-heated buffer solution were added. The experimental setup included four groups: (i) BK group (blank control with no treatment); (ii) RHCA group (supplemented with 3 mg of hyocholic acid (HCA), purity >99%); (iii) RHDCA group (supplemented with 3 mg of hyodeoxycholic acid (HDCA), purity ≥98%); and (iv) RCDCA group (supplemented with 3 mg of chenodeoxycholic acid (CDCA), purity ≥98%). HCA was procured from Sigma-Aldrich (Shanghai, China), CDCA and HDCA were sourced from Chengdu Glip Biotechnology Co., Ltd. (Chengdu, China). To establish anaerobic conditions, all bottles were flushed with N₂ gas, quickly sealed with butyl rubber stoppers and Hungate’s screw caps, and immediately connected to the AGRS-III apparatus using medical-grade transfusion tubing following our previous procedure [3].

### Quantitative bile acid metabolomics analysis

Rumen fluid bile acids were quantified following the procedure described previously [49]. An UHPLC-QE Orbitrap/MS system (Vanquish, Thermo Fisher Scientific) equipped with a Waters ACQUITY UPLC BEH C18 column (150 × 2.1 mm, 1.7 μm particle size) was utilized for the analysis. For assay development, an Orbitrap Exploris 120 mass spectrometer (Thermo Fisher Scientific) was employed. Detailed conditions for UHPLC-QE Orbitrap/MS separation and analysis can be found in our prior study [3].

Based on the definitions of primary and secondary bile acids—considering microbial transformation and modifications—and referencing a previous study [2, 3], the bile acids were classified as follows: primary bile acids: CDCA, ω-muricholic acid (ω-MCA), β-muricholic acid (β-MCA), HCA, cholic acid (CA), CDCA-3-sulfate. Secondary bile acids: isolithocholic acid (isoLCA), lithocholic acid (LCA), 7-ketoLCA, 12-ketoLCA, isoursodeoxycholic acid (isoUDCA), iso-hyodeoxycholic acid (isoHDCA), ursodeoxycholic acid (UDCA), HDCA, 3-epideoxycholic acid (3-epiDCA), deoxycholic acid (DCA). Unclassified bile acids: dehydrolithocholic acid (DHLCA), 6-ketoLCA, dehydrocholic acid (DHCA), 3-DHCA. These classifications are based on whether the bile acids are primary—originating directly from hepatic synthesis—or secondary—formed through microbial transformation in the gastrointestinal tract.

### Metagenomic sequencing and construction of nonredundant gene set

At the end of each *in vitro* rumen fermentation, 2 ml of fermentation mixed solid–liquid phase was collected into DNase-free polypropylene tubes and stored at −80°C until DNA extraction. Total microbial genomic DNA was extracted from these fermentation samples using a cetyltrimethylammonium bromide (CTAB)-based workflow and subjected to quality control before library construction. Briefly, fermentation samples were lysed in CTAB lysis buffer containing lysozyme at 65°C, with incubation in a 65°C water bath and intermittent mixing to ensure sufficient lysis. The lysate supernatant was extracted with phenol (pH 8.0):chloroform:isoamyl alcohol (25:24:1), followed by extraction with chloroform:isoamyl alcohol (24:1). DNA was precipitated with isopropanol at −20°C, collected by centrifugation, washed twice with 75% ethanol, air-dried, dissolved in ddH_2_O, and treated with RNase A at 37°C for 15 min to remove residual RNA. For each qualified sample, 1 μg of genomic DNA was randomly fragmented to ~350 bp using a Covaris ultrasonicator. Sequencing libraries were then constructed through end repair, A-tailing, Illumina adapter ligation, purification, and PCR amplification. After library construction, preliminary quantification was performed using a Qubit 2.0 Fluorometer (Life Technologies, Carlsbad, California, USA), and the insert size distribution was assessed using an Agilent 2100 Bioanalyzer. The effective library concentration was accurately quantified by quantitative polymerase chain reaction (qPCR) to ensure library quality. Qualified libraries were pooled according to their effective concentrations and the required sequencing depth, and paired-end sequencing was performed on the Illumina PE150 platform.

For preprocessing raw data from the Illumina sequencing platform, Readfq (https://github.com/cjfields/readfq) was utilized to obtain clean data for further analysis. The specific steps included: (i) removing low-quality reads (default quality threshold ≤38) that exceeded a certain proportion (default length 40 bp); (ii) eliminating reads with a high percentage of N bases (default length 10 bp); (iii) filtering out reads overlapping with adapters beyond a defined threshold (default length 15 bp). To account for potential host contamination, clean data were subjected to BLAST analysis against the host database (ARS-UI_Ramb_v2.0 *Ovis aries*; NCBI accession: GCF_016772045.1) using Bowtie2 (V.2.2.4) [50] with parameters: --end-to-end, --sensitive, -I 200, and -X 400 [51–53]. Assembly analysis of clean data was performed with MEGAHIT [54] (v.1.1.1) software is used for assembly analysis of Clean Data, with assembly parameter settings: --presets meta-large (--end-to-end, --sensitive, -I 200, -X 400) [52, 55], and Scaftigs without N is obtained by breaking the resulted Scaffolds from the N junction [56]. With the default parameters, MetaGeneMark [57] (http://topaz.gatech.edu/GeneMark/) was then employed for open reading frame (ORF) prediction on Scaftigs (≥500 bp) [51, 58–60], filtering out predictions shorter than 100 nt [55–57, 61, 62]. For the ORF prediction results, CD-HIT [21, 63] is used to eliminate redundancy and obtain the nonredundant initial gene catalogue (the nucleic acid sequences encoded by successive nonredundant genes are called genes) [62], with parameter settings: -c 0.95,-G0,-aS 0.9,-g 1,-d 0 [58, 60]. Clean data from each sample were aligned to the initial gene catalogue using Bowtie2 to calculate the number of reads per gene, with parameters: --end-to-end, --sensitive, -I 200, -x 400 [56, 58]. Genes with reads ≤ 2 in each sample were filtered out to finalize the gene catalogue (unigenes) for subsequent analyses [62]. Gene abundance for each sample was calculated based on the number of aligned reads and gene length, using the formula where *r* represents the number of gene reads and *L* denotes gene length [64–66].

DIAMOND [65] is used to align Unigenes with those in the functional database, with parameter settings: blastp (-e 1e^-5^) [58, 67]. Functional databases include the KEGG database (http://www.kegg.jp/kegg/). From the alignment results of each sequence, the Best Blast Hit results are selected for subsequent analysis [56, 58, 60, 68].

### Functional gene quantification

BileActome HMM profiles were constructed with hmmbuild function in HMMER v3.3.2 [17], and all family models were merged and indexed with hmmpress to generate a searchable profile database. ORFs predicted from the nonredundant gene catalog were queried against the BileActome database using hmmscan function, and significant hits were retained using a stringent threshold (E-value ≤1 × 10^−3^). Coding nucleotide sequences corresponding to the annotated proteins were retrieved from the gene catalog based on protein-to-gene mappings. High-quality paired-end metagenomic reads were aligned to the extracted functional CDSs using Bowtie2 (v2.4.2) [69], and mapping results were used to compute gene-level abundances as RPKM (reads per kilobase per million mapped reads). Gene-level RPKM values were then aggregated by BileActome families to obtain a function-level abundance matrix. For downstream comparative analyses, function-level abundances were converted to relative abundances within each sample and subsequently centered log-ratio (CLR)–transformed.

### Definition of BileActome gene clusters

To characterize genomic co-localization patterns of bile acid metabolic genes, we defined tight gene clusters based on physical proximity of annotated BileActome genes on individual contigs: consecutive BileActome-hit ORFs were assigned to the same “tight cluster” only if they (i) same direction (*require_same_strand* = True), (ii) were separated by, at most, three intervening ORFs (*max_intervening_orfs* ≤ 3), and (iii) had a mean intergenic distance along the ORF path of at most 375 bp (*mean_intergenic_bp* ≤ 375) [70]. Each cluster was then classified solely based on the set of BileActome gene families present in that cluster.

For each cluster, family names were normalized, and presence/absence of each BileActome gene family was recorded. Clusters were first screened for single-family repeats, defined as clusters containing only one unique BileActome family (for example, multiple adjacent *HSDH* genes), which were labeled as “Single-family repeat.”

Remaining clusters were classified using biologically informed gene-set rules. *Bai*-major genes (*BaiB*, *BaiA2*, *BaiCD*, *BaiE*, *BaiF*, *BaiH*) [18] and *Bai*-auxiliary genes (*BaiA1*, *BaiI*, *BaiJ*, *BaiK*, *BaiN*, *BaiO*, *BaiP*) [22, 71], together with the *BaiG* transporter, were used to determine the *Bai*-module structure. A *Bai*-major core was defined as clusters containing at least three *Bai*-major genes. Clusters containing *Bai* genes but not satisfying this criterion were classified as partial *Bai* modules. *HSDH*-only clusters were defined as clusters containing one or more *HSDH* genes but no *Bai* operon genes. Clusters containing only *BSH* genes were classified as *BSH*-only. Remaining mixed configurations were grouped as other mixed clusters. This classification enabled systematic quantification of modular versus complete *Bai*-like structures across the rumen genome collection.

### Definition of microbial bile acid metabolic guilds (operational rules)

Each RHMWGS genome was assigned to a bile acid metabolic guild based on presence/absence of BileActome families. For each family, hmmsearch hits were binarized (present if ≥1 hit). We summarized *Bai* content as (i) *bai_major_count*, the number of *Bai*-major genes present (*BaiB*, *BaiA2*, *BaiCD*, *BaiE*, *BaiF*, *BaiH*), (ii) *bai_aux_count*, the number of *Bai*-auxiliary genes present (*BaiA1*, *BaiI*, *BaiJ*, *BaiK*, *BaiN*, *BaiO*, *BaiP*), and (iii) *has_BaiG*, the presence of the BaiG transporter; *bai_any_count* = *bai_major_count* + *bai_aux_count* + *has_BaiG*. A *Bai*-major core was defined as *bai_major_count* ≥ 3. Genomes were then assigned to mutually exclusive *Bai* guilds: (i) *Bai*-major core + *BaiG* (*BaiG*-positive specialists), (ii) *Bai*-major core without *BaiG* (*BaiG*-negative specialists), (iii) partial *Bai*-module carriers (*bai_any_count* > 0 but no *Bai*-major core), and (iv) no *Bai* module (*bai_any_count* = 0). In parallel, *BSH* capacity (presence of *BSH-G* and/or *BSH-T*) and *HSDH* capacity (number of distinct *HSDH* families present) were computed as auxiliary genome-level features and used for downstream stratification of oxidation/epimerization and deconjugation potential.

### Neighborhood features analyses

To characterize local genomic context beyond tight clusters, we computed neighborhood features for every BileActome hit on each RHMWGS contig. Briefly, for each genome, all hmmsearch hits assigned to the 27 BileActome families were mapped back to their contig coordinates by parsing the corresponding protein identifiers and extracting ORF start/end positions and strand information from the translated protein headers. Within each contig, hit ORFs were sorted by genomic coordinate to define an ORF order index and to enable adjacency-aware distance calculations [72].

For each hit ORF (one row per hit), we quantified nearest-neighbor proximity and local functional density. Intergenic distances between two hits were defined as the base-pair gap between their ORF intervals on the same contig (overlapping intervals were assigned 0). We then recorded the distance to the nearest hit of any family, including whether the nearest neighbor was on the same strand, as well as distances to the nearest hit within each major functional group (*Bai, HSDH*, and *BSH*). In addition, we counted the number of *Bai-, HSDH*-, and *BSH*-group hits within two fixed neighborhood windows (default 10 and 20 kb) around each focal hit, producing per-hit context features that summarize whether a gene is embedded in *Bai*-enriched neighborhoods, co-localized with oxidation–epimerization functions, or isolated from other bile acid genes. For transparency and reproducibility, the window sizes used for these calculations were recorded in the output.

All neighborhood features were exported as tabular files with all parameter settings recorded for full reproducibility. Specifically, the per-hit context table *neighborhood_features* reports genome/contig identifiers, ORF coordinates, and strand, BileActome family and group labels (*Bai/HSDH/BSH*), hmmsearch hit strength including bitscore normalized by amino-acid length (bitscore/aa_len), nearest-neighbor distances (to the nearest hit of any family and to the nearest *Bai/HSDH/BSH*-group hit), and group-specific hit counts within two fixed windows. The neighborhood window parameters are included directly in the table as *window_bp* = 10 000 and *window2_bp* = 20 000 (i.e. counts of *Bai*/*HSDH*/*BSH* hits within 10 and 20 kb of each focal hit) [72].

### Statistical analysis

Abundances of microbial KOs and BileActome compared using Student’s *t-*test in SPSS 26.0 (IBM, NY, USA) (*P*-value <.05), indicating statistical significance. For targeted bile acid metabolomics, |log₂fold change| > 2, along with variables assessed by Student’s *t-*test with a *P*-value <.05, were identified as differential bile acids.

## Results

### Overview of the BileActome framework and application

**Figure 1:**
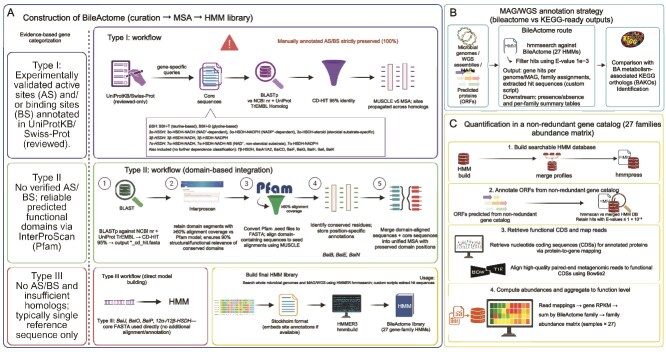
BileActome enables high-resolution annotation of microbial bile acid metabolism. A workflow illustrating BileActome construction (A), and scalable application of BileActome to isolate genomes, metagenome-assembled genomes, and gene catalogs (B, C). Created in BioRender. Zhang, B. (2026) https://BioRender.com/klu4nvq

BileActome adopts an evidence-tiered design that explicitly matches model construction strategy to the strongest available biochemical/structural evidence for each gene family. Type I families are anchored on UniProt/Swiss-Prot reference sequences with experimentally curated active sites and/or ligand-binding sites, so that function-defining constraints are preserved as homologous diversity is incorporated. Type II families correspond to experimentally verified enzymes but lacking curated catalytic-site evidence; for Type II, BileActome centers model construction on conserved domains [73, 74] and emphasizes high-confidence domain segments to balance sensitivity for divergent homologs with control of spurious functional assignment [75, 76]. Type III families were defined as lower-evidence close-homolog detection models for rare bile acid metabolic genes for which only single or very limited references are available and neither catalytic sites nor conserved domains have been characterized. These models were retained to enable systematic detection of close homologs while explicitly indicating their lower evidence strength relative to Type I and Type II models (Fig. 1A).

Beyond evidence tiering, BileActome is designed to resolve biochemically meaningful subtypes that are commonly conflated in generic resources. In particular, *BSH* and *HSDH* are widespread bile acid–modifying enzymes [12, 13, 15], yet each family comprises distinct subtypes with different biochemical consequences; treating them as undifferentiated groups obscures mechanistic variation [14, 16]. BileActome therefore separates *BSH-G* and *BSH-T* based on established substrate specificity (glycine- vs taurine-conjugated bile salts) [14, 77]. For *HSDH*s, BileActome resolves reactions by position (C3/C7/C12) and distinguishes major variants by cofactor usage (NAD^+^ vs NADP^+^) and substrate preference (steroidal vs nonsteroidal), which are governed by conserved motifs and structural determinants [16, 78, 79]. Encoding these validated subtype distinctions directly into the model architecture allows BileActome outputs to map to concrete enzymatic functions rather than broad pan-family membership, strengthening links between gene inventories and bile acid transformation chemistry.

BileActome is released as an open-access, reusable resource, together with scripts for reproducible deployment. It supports two primary application modes. For genome-resolved analyses (isolate genomes, MAG, WGS collections), predicted proteins are queried against the BileActome, and hits are summarized per genome to generate presence–absence matrices, copy-number profiles, and Boolean indicators for downstream phylogenetic, cluster, or guild analyses. For community-level functional profiling, predicted ORFs from a nonredundant metagenomic gene catalog are scanned against the same library to extract bile acid–related genes; corresponding nucleotide sequences can then be used for read-mapping–based quantification to construct gene- or family-level abundance matrices across samples, which can be transformed into compositional formats for downstream comparative analyses. Because all models are built under a consistent evidence-tiered framework and standardized thresholds, BileActome outputs are designed to be directly comparable across studies and readily extensible as new bile acid enzymes are experimentally characterized (Fig. 1B and C).

### Definition-level comparison of BileActome families and KEGG bile acid knockouts

**Figure 2:**
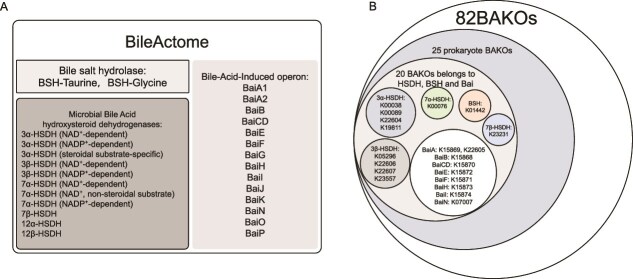
Comparison of gene sources and functional scope between BileActome and the KEGG bile acid KO (BAKO) system. (A) Composition of the BileActome library, comprising 27 experimentally supported microbial bile acid metabolism gene families organized into three functional pillars: bile salt hydrolases (*BSH*; taurine- and glycine-specific), bile acid–inducible operon genes (*BaiA–P*), and microbial hydroxysteroid dehydrogenase (*HSDH*) with explicit cofactor and substrate specificities. (B) Conceptual organization of the KEGG bile acid–related KO (BAKO) system, showing that most bile acid–associated KOs are not prokaryote-specific and include host (eukaryotic) enzymes, with only a subset mapping to microbial *BSH*, *Bai*, and *HSDH* functions. The comparison highlights the microbiome-specific focus and biochemical resolution of BileActome relative to the broader, mixed-origin BAKO collection.

BileActome is organized as 27 experimentally supported, prokaryote-derived microbial gene families spanning *BSH*, *BaiA–P*, and microbial *HSDH*s, with explicit subtype structure and redundancy control, thereby providing a microbiome-focused and biochemically resolved annotation framework (Fig. 2A). In contrast, KEGG provides a curated KO framework and includes a set of bile acid–related KO (BAKO) as we defined in a previous study [3]; however, many of these entries originate from eukaryotic-only enzymes or eukaryote-dominated contexts that are not directly relevant to microbial bile acid metabolism, so in this study, we therefore used a prokaryote-focused subset of 20 BAKOs. The 20 prokaryote-focused BAKOs were selected from the 82 BAKOs defined in our previous study based on the “Taxonomy Mapping-KEGG Organisms in Taxonomic Ranks” information obtained for each KO from the KEGG official website (https://www.kegg.jp/). KOs with prokaryotic taxonomic representation were retained, whereas KOs represented only by eukaryotic taxa were excluded (Supplementary Table S3). In addition, KO-level entries often lack explicit biochemical resolution for key reaction variants, resulting in conflation of mechanistically distinct transformations (e.g. cofactor- or substrate-specific isoforms) (Fig. 2B, Supplementary Table S3). This conceptual distinction is reflected across several key dimensions (Table 1). These definition-level properties are intended to improve interpretability of microbial bile acid metabolic potential from genomes and gene catalogs.

**Table 1:** Conceptual and functional comparison between the KEGG bile acid KO (BAKO) system and BileActome.

Category	KEGG Bile Acid KO System	BileActome
Data source	Curated KEGG Orthology (KO) entries; ~82 bile acid–related KOs (BAKOs)	27 experimentally validated genes (*BSH*, *BaiA–P*, *3α/3β/7α/7β/12α/12β-HSDHs*)
Prokaryotic relevance	Only 25 prokaryotic KOs; many are eukaryote-only	All 27 genes derived from prokaryotic genomes
Functional completeness	20 prokaryotic BAKOs covering *BSH*, *Bai*, limited *HSDHs* (3α/3β/7α/7β only)	Complete cascade including *12α/12β-HSDHs*, and subtype-specific *3α/3β/7α-HSDHs*
Subtype/cofactor specificity	No differentiation among NAD^+^/NADP^+^ or substrate variants	Distinguishes -Taurine, -Glycine, NAD^+^-, NADP^+^-, steroidal-, and nonsteroidal-specific isoforms

BileActome models were built from curated reference sequences followed by systematic homology expansion and redundancy-controlled de-replication, yielding standardized, reusable functional annotation database with transparent evidence tiering and consistent operating thresholds. By contrast, BAKO entries inherit KEGG’s broader KO aggregation logic, and sequence counts per KO reflect heterogeneous provenance and curation scope rather than a microbiome-specific redundancy-controlled modeling strategy. Collectively, BileActome and BAKO provide complementary annotation perspectives: BAKO offers a broad KEGG-based framework for pathway-level comparison, whereas BileActome provides a subtype-resolved, prokaryote-focused framework for finer annotation of microbial bile acid metabolic potential, thereby motivating the genome-scale and abundance-based benchmarks reported in the following sections (Fig. 2A and B, Table 2).

**Table 2:** Comparison of source sequence counts used to construct BileActome and KEGG bile acid KOs (BAKOs).

Gene	BileActome	BAKO
*BSH*	565	569
*BaiA1*	150	2
*BaiA2*	82	10
*BaiB*	92	326
*BaiCD*	6	6
*BaiE*	46	3
*BaiF*	332	6
*BaiG*	4	0
*BaiH*	11	6
*BaiI*	2	2
*BaiJ*	1	0
*BaiK*	3	0
*BaiN*	67	2
*BaiO*	1	0
*BaiP*	1	0
*3α-HSDH*	1341	1835
*3β-HSDH*	800	195
*7α-HSDH*	81	1210
*7β-HSDH*	316	5
*12α-HSDH*	1	0
*12β-HSDH*	2	0

### Community-level responses and phylogeny-structured distribution of bile acid metabolic potential

**Figure 3:**
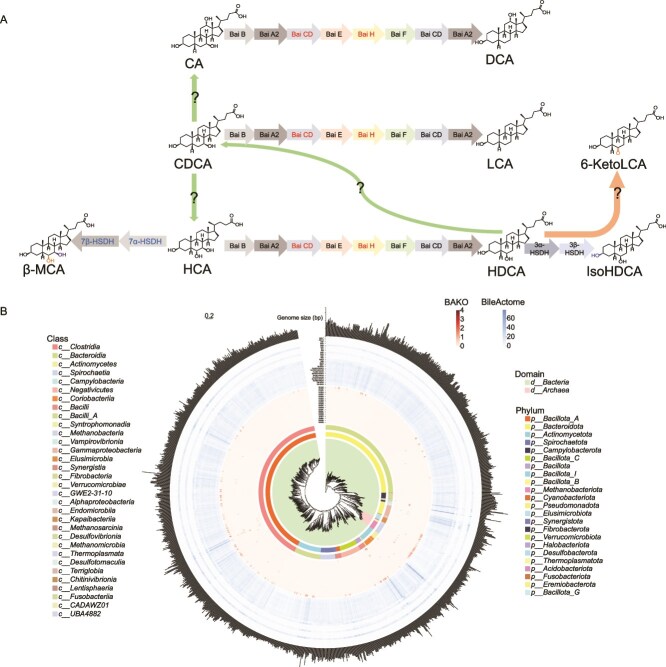
BileActome-enabled reconstruction of bile acid transformation pathways and genome-resolved functional landscapes in the rumen microbiome. (A) Conceptual reconstruction of microbial bile acid conversion routes supported by BileActome, highlighting alternative and modular organizations of main *Bai* gene clusters (*BaiB–BaiA2–BaiCD–BaiE–BaiH–BaiF*) and their coupling with *HSDH*-mediated oxidation-epimerization interconversions. Gene families showing significant or trending upregulation or downregulation are indicated in the schematic *(P*-value <.1), whereas gene families with no trending difference are shown separately (*P*-value >.1). The schematic illustrates that 7α-dehydroxylation modules (*Bai*) can occur in partial, rearranged, or dispersed genomic contexts, and may interface with *HSDH*-catalysed epimerization steps across genomes, consistent with community-assembled bile acid transformation rather than a single conserved operon. Curved arrows indicate putative cross-genome functional coupling among pathway segments, and question marks denote reaction links not encoded within a single genome. (B) Phylogeny-aligned heatmaps comparing 20 KEGG bile acid KOs (BAKOs) and BileActome family annotations across the 1693 rumen high-quality metagenome-assembled genomes and whole-genome sequences (RHMWGS). BAKO annotations are sparse and lineage-restricted, whereas BileActome reveals a broad, structured distribution of bile acid metabolic capacities across the rumen tree, consistent with widespread but modular community-level encoding of bile acid deconjugation, oxidation-epimerization modification, and dehydroxylation functions.

Using the *in vitro* rumen fermentation dataset, we first reconstructed treatment-specific bile acid transformation routes for RHCA vs BK, RHDCA vs BK, and RCDCA vs BK based on the differential bile acids identified by targeted bile acid metabolomics (Supplementary Fig. S1A–C). We then integrated these treatment-specific routes with the corresponding BileActome family-level changes derived from the metagenomic nonredundant gene catalog, and summarized them as a consolidated HCA-, HDCA-, and CDCA-associated bile acid biotransformation map (Fig. 3A). Families showing consistent directional changes across contrasts [red and blue indicate significant or trending increases and decreases, respectively, in BileActome family abundance in the relevant treatment-versus-BK comparison (*P*-value <.1), whereas black indicates no trending difference (*P* value >.1)], thereby highlighting cross-condition reproducibility at the enzyme-family level. In this integrated view, the most reproducible signals center on the *Bai* module, with recurrent differential responses in BaiN, which may contribute to the reductive steps of Bai-mediated transformations, and additional Bai components showing repeated support across treatments (*BaiG, BaiCD, BaiH*, and condition-specific *BaiB*), consistent with *Bai*-mediated transformations forming a core axis of community response to bile acid perturbation (Supplementary Fig. S1).

We summarized BAKOs using the same compositional preprocessing used for BileActome profiles (pseudocount handling and CLR transformation applied to the full KO composition prior to subsetting BAKO, we selected 20 BAKOs, which originates from prokaryotes and functions as *BSH*, *Bai*, and *HSDH* (Fig. 2B, Supplementary Table S3). Consistent with the pathway (Fig. 3A), KO-level differential signals are most evident for *Bai*-associated BAKOs: *BaiB* (K15868) shifts significantly in RHCA vs BK (*P*-value <.05), and *BaiH* (K15873) shifts significantly in RCDCA vs BK (*P*-value <.05), whereas no BAKO reaches significance for RHDCA vs BK (Supplementary Table S4).

To benchmark detection at the genome scale, a phylogeny-aligned comparison between the 20 BAKOs and BileActome across the 1693 RHMWGS tree was conducted (Fig. 3B). The circular display integrates (i) an inner taxonomy annotation (domain, phylum, and class rings), (ii) two intensity tracks summarizing bile acid-related signals per genome under KEGG and BileActome, and (iii) an outer genome-size track. RHMWGS is dominated by bacteria (1639/1693; 96.8%) with a smaller archaeal component (54/1693; 3.2%). At the phylum/class level, the dataset is primarily composed of *Bacillota_A*/*Clostridia* (701 genomes) and Bacteroidota/*Bacteroidia* (436/435 genomes), with additional contributions from *Bacillota_I*/*Bacilli_A* (119 genomes), *Bacillota_C*/*Negativicutes* (82 genomes), *Actinomycetota* (68 genomes; including *Coriobacteriia* and *Actinomycetes*), *Spirochaetota*/*Spirochaetia* (63/62 genomes), and *Pseudomonadota*/*Gammaproteobacteria* (59/47 genomes), alongside *Methanobacteriota*/*Methanobacteria* (39 genomes) and other lower-abundance phylum. Under the KEGG workflow, only 222/1693 genomes (13.1%) carry at least one detectable BAKO hit in this comparison, and the per-genome BAKO signal is frequently zero across large regions of the tree. In contrast, BileActome yields a broad signal across RHMWGSs: all 1693/1693 genomes contain at least one BileActome family hit and carry many BileActome families simultaneously (median 18/27 families present per genome, interquartile range 17–19). This broad detection should be interpreted considering the family breadth of BileActome rather than as evidence that every genome encodes a complete bile acid transformation pathway. BileActome includes widely distributed BSH and HSDH families associated with deconjugation and oxidation/epimerization potential, whereas complete or near-complete Bai repertoires remain much more restricted.

### Genome-resolved distribution, sequence diversity, and modular organization of bile acid metabolic genes in the rumen microbiome

**Figure 4:**
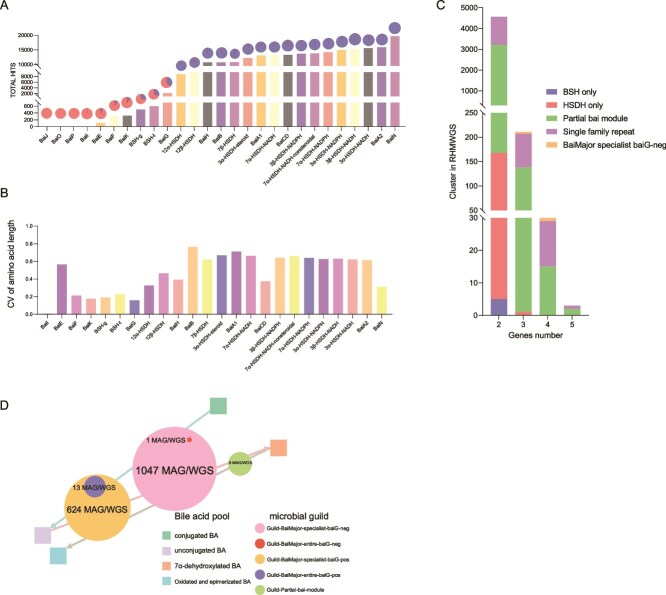
Genome-resolved distribution, sequence diversity, and modular organization of bile acid metabolic genes in the rumen microbiome. (A) Total hits of each BileActome family across the 1693 rumen high-quality metagenome-assembled genomes and whole-genome sequences (RHMWGS). The small pie chart above each BileActome family shows the proportions of RHMWGS genomes with at least one detected hit and without a detected hit for that family. (B) Amino acid sequence length distributions of rumen-derived homologs for each BileActome family. (C) Cluster-level organization of BileActome genes based on standardized neighborhood definitions (intergenic distance and ORF proximity). (D) Genome-level guild composition of bile acid metabolic functions across RHMWGS. Microbial bile acid metabolic guilds were assigned based on the presence/absence of BileActome families in each RHMWGS genome. Bai-major genes were defined as *BaiB*, *BaiA2*, *BaiCD*, *BaiE*, *BaiF*, and *BaiH*; Bai-auxiliary genes were defined as *BaiA1*, *BaiI*, *BaiJ*, *BaiK*, *BaiN*, *BaiO*, and *BaiP*; and *BaiG* was considered separately as the *BaiG* transporter. Genomes with at least three Bai-major genes were considered to contain a Bai-major core and were further divided into *BaiG*-positive and *BaiG*-negative specialists according to *BaiG* presence. Genomes with Bai genes but without a Bai-major core were classified as partial Bai-module carriers, whereas genomes without Bai genes were classified as no *Bai* module. *BSH* and *HSDH* capacities were used as auxiliary features to interpret deconjugation and oxidation/epimerization potential.

Screening the 1693 RHMWGS genomes revealed a structured distribution of bile acid metabolic potential (Fig. 4A). *HSDH*-related families were the most abundant across genomes, followed by *BSH* families, whereas genomes carrying complete *Bai* repertoires were comparatively rare, indicating that deconjugation and oxidation-epimerization modification capacities are more broadly distributed than dehydroxylation.

The rumen-derived homologs supporting these annotations are highly heterogeneous in sequence level (Fig. 4B). Across BileActome families, amino-acid sequence length distributions show broad dispersion, and several families exhibit substantial intrafamily variability, indicating that similar biochemical functions in rumen microbiomes are not necessarily reflected by globally similar sequences. This degree of heterogeneity limits the effectiveness of direct similarity-based discovery and reinforces the need for profile-based models that capture conserved functional constraints and structural determinants rather than relying on global sequence identity alone [10, 80–82].

To answer whether bile acid genes in rumen genomes are typically organized into tight neighborhoods, we applied a standardized “tight neighborhoods” definition that groups BileActome hits into local neighborhoods on the same contig based on ORF adjacency and intergenic distance constraints. Each resulting cluster was then classified solely by its BileActome family composition, enabling a genome-wide survey of whether *Bai* genes tend to co-localize as compact modules or appear as fragmented neighborhoods (Fig. 4C). In practice, most clusters did not correspond to complete *Bai* operons. Instead, clusters were dominated by partial *Bai* subsets (often lacking a full *Bai*-major complement), and mixed neighborhoods were common in which *Bai* genes co-occurred with *HSDH* and/or *BSH* loci within the same local region. Conversely, fully self-contained *Bai* neighborhoods were comparatively infrequent, indicating that even when *Bai* genes are present, they are often embedded within broader, heterogeneous bile acid–modification contexts rather than organized as a single compact unit. Together, these patterns support a modular organization in which functionally linked steps are frequently encoded across fragmented genomic neighborhoods, consistent with earlier evidence that bile acid metabolic gene organization departs from textbook operon structures [70].

Finally, collapsing neighborhood and gene-inventory patterns into genome-level guilds highlighted an ecological organization consistent with auxotrophy-like, community-assembled bile acid metabolism (Fig. 4D). Guilds were defined using explicit Boolean rules on BileActome inventories—summarizing *Bai* completeness via *Bai*-major membership (*BaiB*/*BaiA2*/*BaiCD*/*BaiE*/*BaiF*/*BaiH*), *Bai*-auxiliary components, and the *BaiG* transporter—and assigning genomes to mutually exclusive categories representing *Bai*-major core carriers, *Bai*-major specialists, or partial *Bai*-module carriers, with *BSH*/*HSDH* functions treated as additional capacities layered onto these *Bai*-defined states. These guilds were then interpreted through an abstract bile acid pool × reaction scaffold [83], which does not assume that complete conversion pathways reside within single genomes but instead explicitly represents successive chemical transformations as distributed across functional guilds. Guilds-scale studies suggest that function of microbial bile acid metabolism in the rumen can be maintained through functional redundancy [84].

### Neighborhood context of BileActome hits in rumen high-quality metagenome-assembled genomes and whole-genome sequences

Using the per-hit neighborhood feature (Supplementary Table S5), we quantified local functional density around each BileActome-positive ORF by counting nearby hits within fixed windows (10 and 20 kb) [72]. After collapsing multi-family assignments to a single representative hit per ORF, we obtained ~5.7 × 10^^4^ unique BileActome-positive ORFs. Neighborhood counts revealed clear and nonrandom proximity patterns across functional groups. For *Bai* hits, local *Bai* density was common: 60.3% of *Bai* hits had at least one additional *Bai* hit within 10 kb (*n_Bai_within_10kb* > 0), increasing to 66.1% within 20 kb (*n_Bai_within_20kb* > 0). *Bai* hits also frequently co-occurred with nearby oxidation–epimerization capacity: 21.1% of *Bai* hits had at least one *HSDH* hit within 10 kb (*n_HSDH_within_10kb* > 0), increasing to 25.8% within 20 kb (*n_HSDH_within_20kb* > 0). By contrast, *BSH* proximity around *Bai* hits was rare (≤0.4% even within 20 kb; *n_BSH_within_10kb*/*20 kb* > 0), indicating that deconjugation loci are generally not embedded within *Bai*-dense neighborhoods.

For *HSDH* hits, neighborhoods were even more densely populated: 90.3% of *HSDH* hits had another *HSDH* hit within 10 kb (*n_HSDH_within_10kb* > 0) and 90.9% within 20 kb (*n_HSDH_within_20kb* > 0), consistent with widespread local clustering of oxidation–epimerization functions. Moreover, 80.9% of HSDH hits had at least one Bai hit within 10 kb (n_Bai_within_10kb > 0), rising to 83.5% within 20 kb (n_Bai_within_20kb > 0), indicating frequent Bai–HSDH proximity at the contig scale. This pattern may reflect functional coupling between Bai-mediated transformations and HSDH-mediated oxidation/epimerization, shared genomic contexts of bile acid–related genes, or lineage-specific enrichment of these gene families.

In contrast, *BSH* hits exhibited a more independent neighborhood signature. Only 17.9% (10 kb) and 31.0% (20 kb) of *BSH* hits had nearby *Bai* hits (*n_Bai_within_10kb/20 kb* > 0), and only 6.8% (10 kb) and 11.6% (20 kb) had nearby *HSDH* hits (*n_HSDH_within_10kb/20 kb* > 0). However, *BSH* hits strongly co-occurred with other *BSH* hits: 83.1% of BSH hits had at least one additional *BSH* hit within 10 kb and 83.6% within 20 kb (*n_BSH_within_10kb*/*20 kb* > 0). Together, these window-based neighborhood features indicate that *Bai* and *HSDH* functions frequently reside in proximity, whereas *BSH* loci are typically distributed more independently.

## Discussion

KEGG provides a useful baseline for comparative analysis because it offers a widely used and standardized KO framework that supports consistent genome- and metagenome-level summaries across studies. However, in the context of bile acid metabolism, its broad classification system has clear limitations for microbiome-scale analysis [7]. BileActome was developed to address this gap by providing a microbiome-focused, subtype-resolved annotation framework in which gene families are explicitly defined for prokaryotic bile acid metabolism. By distinguishing functionally important subtypes, particularly within *BSH* and *HSDH* families, and by using a controlled and reproducible curation strategy, BileActome supports more precise interpretation of reaction steps and community-level organization [15, 84].

In the non-redundant gene-catalog comparisons, the current study using three individual bile acid treatments (HCA, HDCA, and CDCA) produced reproducible shifts centered on the *Bai* module, with *BaiN* significant across all three contrasts and additional components repeatedly supported (*BaiG, BaiCD, BaiH*, and condition-specific *BaiB*), consistent with *Bai*-mediated transformations forming a core axis of community response to bile acid stress. Although *BaiN* lacks direct experimental validation, our previous rumen-scale analyses identify it as among the most abundant *Bai*-associated components [3], suggesting a potentially important role in bile acid stress tolerance or pathway throughput that warrants targeted validation. At the genome scale, BileActome showed that bile acid metabolic capacity is broadly distributed across RHMWGS and appears to be functionally redundant, suggesting that this activity is maintained by many taxa rather than a few specialized lineages. In contrast, KEGG-based summaries were sparse and uneven across the phylogeny, which could give the misleading impression that bile acid metabolism is limited to a narrow group of core taxa [84, 85].

The genome-resolved landscape also revealed a clear hierarchy of functional distribution. HSDH-related families were the most widespread, followed by *BSH* families, whereas genomes carrying complete *Bai* repertoires were relatively uncommon. This pattern suggests that bile acid metabolism in the rumen is distributed across multiple microbial genomes and phylogenetic groups rather than concentrated in a few genomes carrying complete pathways. These findings also suggest a modular organization of bile acid transformation in the rumen. Key steps, including BSH-mediated deconjugation, HSDH-mediated oxidation and epimerization, and Bai-associated transformations, may operate as partially independent functional modules. As a result, different microbial taxa are likely to contribute distinct components of the overall bile acid transformation network [83, 86, 87]. Together with recent ruminant studies linking gastrointestinal microbiomes to host metabolic adaptation, these results highlight the broader value of function-resolved microbiome resources for understanding ruminant gastrointestinal ecosystems [88, 89].

Neighborhood analysis further showed that bile acid metabolic genes are usually not arranged in compact, pathway-complete operons. Although local proximity among *Bai* genes or among *HSDH* genes was sometimes observed, most BileActome hits were dispersed, and *BSH* loci showed limited co-localization with *Bai* or *HSDH* genes. This independent neighborhood signature of BSH loci suggests that BSH-mediated deconjugation may function as a broadly distributed entry module that generates free bile acids for subsequent microbial transformations without requiring local linkage to downstream Bai or HSDH genes. This has an important practical implication: in the rumen microbiome, genomic proximity alone is not a reliable way to identify new bile acid metabolic genes. Approaches based mainly on adjacent gene context are therefore likely to miss many functionally relevant genes.

Overall, BileActome provides an evidence-based, subtype-resolved, and reproducible framework for analyzing microbial bile acid metabolism from genomes and gene catalogs. Its outputs support interpretation at multiple levels, from individual gene families to local genomic context and genome-level guild structure. As knowledge of bile acid metabolism continues to expand, further refinement of subtype definitions and incorporation of new experimental evidence will improve functional classification. BileActome was designed to support this ongoing development through versioned updates and systematic expansion, so that newly experimentally characterized bile acid enzymes can be incorporated to update family boundaries, add subtype definitions, and revise evidence tiers. Although the present study demonstrates BileActome primarily in the rumen microbiome, the resource itself is not limited to this ecosystem. Because the BileActome library was constructed from public and literature-derived microbial bile acid gene sequences rather than rumen-specific sequences alone, it may also be useful for studying microbial bile acid metabolism in other mammalian host-associated microbiomes. Future applications across additional host species and gastrointestinal niches will help further evaluate their transferability and refine family boundaries as new experimentally characterized enzymes become available.

## Supplementary Material

Supplementary_material_ycag205

## Data Availability

Shotgun metagenomic sequencing data have been deposited in NCBI under the accession number PRJNA1415377. The MAG sequences are also accessible under the accession number PRJNA1415377. RHMWGS is available in https://doi.org/10.6084/m9.figshare.31386229.

## Code availability

The BileActome code repository is publicly available at https://github.com/1362996609zby/Bile_acid_v2. The repository contains the scripts used for BileActome database construction, genome-resolved annotation of rumen MAG/WGS datasets, and community-level profiling based on non-redundant metagenomic gene catalogs.

## Notes

Additional address: Sanya Institute of China Agricultural University, 9th–11th Floors, Innovation and Research Valley Teaching Area, Yazhou Bay Science and Technology City, Yazhou District, Sanya, Hainan, China
