# Supplementary material for: BileActome reveals community-assembled bile acid metabolism in the rumen microbiome
Source: ISME Commun. 2026 Jul 14;6(1):ycag205. doi: 10.1093/ismeco/ycag205 (PMC13477222; doi:10.1093/ismeco/ycag205)
Supplement: Supplementary file 1 [file supplementary_material_ycag205.zip › BYZ_Supplementary_Figure_-_3.17_ycag205.pdf]

Supplementary Figure

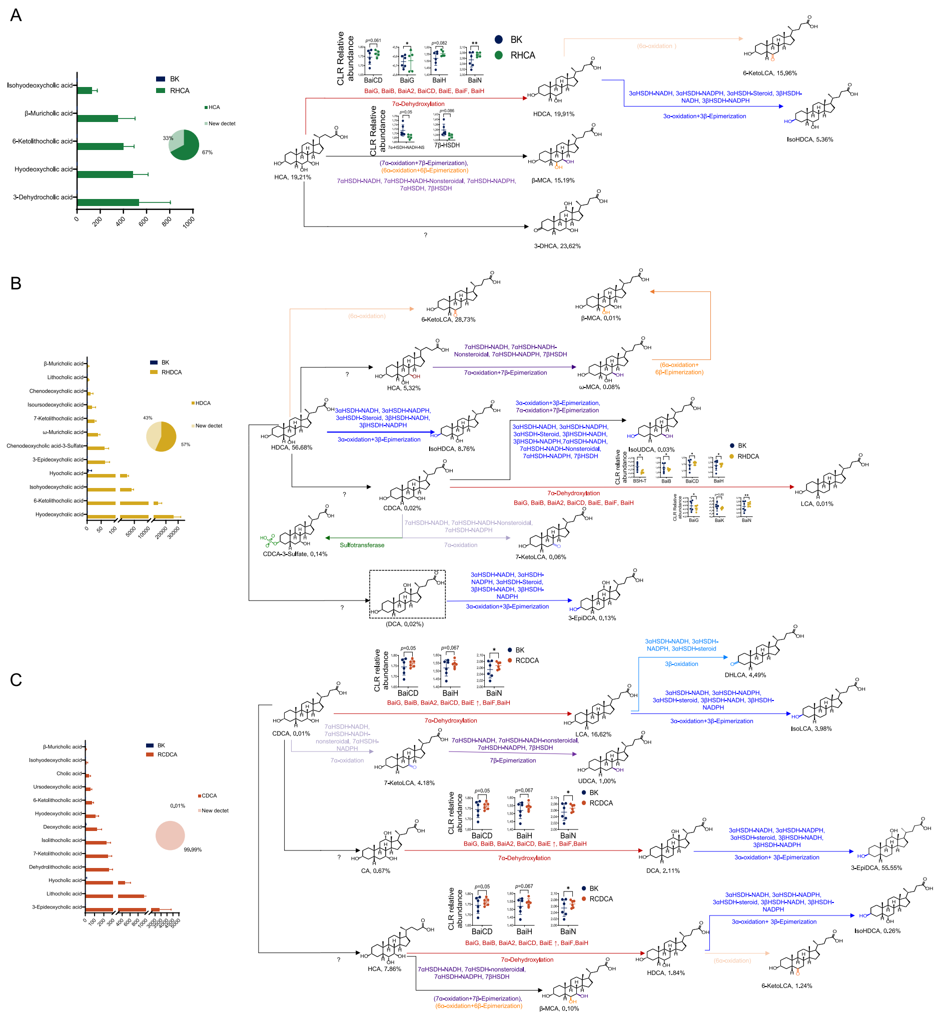

Supplementary Fig. S1. Substrate-specific bile acid transformation routes and associated BileActome gene-family responses in the *in vitro* rumen fermentation. Results for RHCA **(A)**, RHDCA **(B)**, and RCDCA **(C)** treatments. For each condition, the left panels show bile acids that were significantly altered relative to the blank control (BK) ($P < 0.05$, $|\log_2\text{fold change}| > 2$). The right panels depict inferred bile acid transformation routes reconstructed from the observed differential bile acids and established biochemical pathways, with the relative contribution of each bile acid indicated as the percentage of total bile acids. Bar plots overlaid on the pathways indicate significantly altered or trend-level BileActome gene families implicated in each reaction step. Bars represent mean ± s.d. $^{*}P < .05$, $^{**}P < .01$.
